# Is everyone a mix of straight and gay? A social pressure theory of sexual orientation, with supporting data from a large global sample

**DOI:** 10.3389/fpsyg.2023.1187377

**Published:** 2023-07-11

**Authors:** Robert Epstein, Hongyu Wang, Vanessa R. Zankich

**Affiliations:** American Institute for Behavioral Research and Technology, Vista, CA, United States

**Keywords:** sexual orientation, social pressure theory of sexual orientation, Epstein sexual orientation inventory, mean sexual orientation, sexual orientation range, sexual orientation continuum

## Abstract

Sigmund Freud, Alfred Kinsey, E.O. Wilson, and others have suggested that social pressure suppresses natural tendencies for humans to express bisexuality, the apparent norm for one of our two closest genetic relatives, the bonobo. An analysis of data obtained from a new online sample of 1,150,938 people in 215 countries and territories (63.9% from the United States, United Kingdom, and Canada) who completed the English version of a validated questionnaire of sexual orientation lends support to this idea. A histogram of scores from 0 (exclusive opposite-sex inclinations) to 18 (exclusive same-sex inclinations) forms a near-normal distribution. Although this distribution was likely caused to some extent by sampling bias, it may also reflect the unusual honesty people show when taking online tests anonymously, as an increasing body of evidence demonstrates. We present a formal mathematical expression of a social pressure theory of sexual orientation, along with empirical evidence and computational explorations that support the theory. We also present an analysis of the new data set. Among other findings: sexual orientation labels corresponded to broad, skewed, overlapping distributions of scores. Self-labeled gays/lesbians and, to a greater extent, self-labeled straights, reported that the larger the mismatch between their sexual orientation label and their actual sexual inclinations, the more distress they felt regarding their sexual orientation, a finding that is predictable from cognitive dissonance theory. Educating the public about the true nature of sexual orientation might quell the often rancorous public debates on this topic, as well as give comfort to a large number of mislabeled people.

## Introduction

Humans have a strong tendency to categorize phenomena that are in fact continuous in their physical characteristics (Jorde and Wooding, 2004; Culotta, 2012). When should scientists set aside category labels in favor of a continuum model that more accurately describes the phenomenon of interest? This issue has been debated in various scientific fields for at least a century and is still of concern today, in part because of an obvious advantage that continuous variables have over categorical ones. Categories often exist on nominal or ordinal scales of low resolution (male/female, red/blue/yellow), whereas continuous variables tend to make precise measurement possible, facilitating the development of predictive, quantitative models.

Mathematical aspects of continua were discussed at length in *Science* in an essay by Luce and Narens (1987), in which they concluded, “continuous variables are the correct kind of idealization for many, if not most, of the ordered empirical situations encountered in science.” Appropriately, a shift away from categorical thinking has occurred to some extent in many scientific disciplines as they have matured over time, among them botany (Curtis, 1951; Daubenmire, 1966; Vogl et al., 1966), population genetics (Parra et al., 2004; Shriver et al., 2005; de la Mettrie et al., 2007), genomic medicine (Pittman et al., 2004; Willard et al., 2005), gender studies (Hanson, 2000), quantum mechanics (Sachs, 1999), paleontology (Elgin, 1999), and sociology (Wong, 1997).

Sexual orientation, however, is still largely viewed as a categorical phenomenon. It has been 75 years since Alfred Kinsey and his colleagues first published their groundbreaking book on sexual behavior (Kinsey et al., 1948), yet their insights into the continuous and somewhat dynamic nature of sexual orientation (SO) are still not widely accepted (Drucker, 2010). Based on interviews with more than 6,000 people, Kinsey and colleagues concluded that “It is a fundamental of taxonomy that nature rarely deals with discrete categories…. The living world is a continuum in each and every one of its aspects” (Kinsey et al., 1948), and this view was echoed in 2008 in a public statement endorsed by the American Psychological Association and 12 other professional organizations (Just the Facts Coalition, 2008). Nevertheless, many people still generally assume that someone is either “straight,” “gay,” “lesbian,” or, perhaps, “bisexual,” (Bailey et al., 2016) with the vast majority of people adopting the most socially accepted of these labels – “straight,” no matter what their actual sexual inclinations (Bostwick et al., 2010; Herbenick et al., 2010, 2017; Joloza et al., 2010; Epstein et al., 2012; Ward et al., 2014; Copen et al., 2016). Because continuous phenomena do not lend themselves to categorical labeling, it has lately become popular to invent more and more labels describing subtle differences in sexual orientation and gender; we are aware of 36 different sexual orientation labels at this writing (Zambon, 2022; *cf.* Polestar LGBT+ Community Center, 2018).

A 2012 study with 17,785 participants in 48 countries provided clear support for Kinsey’s continuum concept, finding that scores on a questionnaire that measured self-reported sexual attractions, fantasies, and behaviors were distributed smoothly along a continuum from exclusive opposite-sex (OS) to exclusive same-sex (SS) inclinations (Epstein et al., 2012), and other studies have reported similar findings (Ellis et al., 1987; Haslam, 1997; Sell, 1997; Kraemer et al., 2006; Thompson and Morgan, 2008; Chivers et al., 2010; Vrangalova and Savin-Williams, 2012; Epstein and Robertson, 2014). Because actual sexual inclinations are so variable, SO is perhaps best described by two numbers: mean sexual orientation (MSO), a measure of where an individual’s inclinations are centered on the sexual orientation continuum, and sexual orientation range (SOR), an interval around the MSO which suggests how much flexibility one has in expressing one’s SO (Epstein et al., 2012). Like MSO, SOR has been found to vary along a continuum, with some people having little flexibility and others considerably more (Vrangalova and Savin-Williams, 2010; Epstein et al., 2012; Geary et al., 2017). In the present study, we examined this issue using a large international sample and also asked a question of practical importance: what price is paid when one’s SO label does not match one’s actual sexual inclinations?

In many societies, people are expected to call themselves “straight” and to act on OS inclinations exclusively (Mendos et al., 2020). In some societies, people are allowed in varying degrees to describe themselves in other ways – in the US, by using the labels “gay” or “bisexual,” for example. Once one adopts a label, one is also sometimes discouraged from switching to a different one, even if one’s sexual inclinations are changing (American Psychological Association Task Force, 2009). Implicit in such practices is the assumption that SO labels accurately distinguish discrete and fixed categories of sexual inclination. But two common research findings – first, that SO lies on a continuum (Ellis et al., 1987; Haslam, 1997; Sell, 1997; Kraemer et al., 2006; Thompson and Morgan, 2008; Chivers et al., 2010; Epstein et al., 2012; Vrangalova and Savin-Williams, 2012; Epstein and Robertson, 2014; Savin-Williams, 2014; *cf.* Gangestad et al., 2000; Savin-Williams and Vrangalova, 2013; Norris et al., 2015) and second, that there is some flexibility and fluidity in the way people express their SO over time (Chung and Katayama, 1996; Garnets and Peplau, 2000; Rosario et al., 2006; Diamond, 2008; Ott et al., 2011; Ross et al., 2012; Savin-Williams et al., 2012; Savin-Williams and Vrangalova, 2013; Norris et al., 2015; Morandini et al., 2021; Xu et al., 2021; Srivastava et al., 2022a,b) – suggest that for many people SO labels are out of sync with actual sexual inclinations. How big is this mismatch, and what consequences does it have?

When people label themselves a certain way (say, Republican) but behave in a way that is inconsistent with that label (say, by frequently voting for Democrats), how does that discrepancy play out? Research on cognitive dissonance suggests that a discrepancy of this sort is often unstable (Festinger, 1957; Festinger and Carlsmith, 1959; Cooper, 2007) and that the larger the discrepancy, the more distress people experience (Elliot and Devine, 1994). In the present study, we sought not only to quantify the mismatch between SO labels and actual sexual inclinations but also to measure its possible emotional consequences.

We offer two possible interpretations of our main findings, neither of which, we argue, can be ruled out entirely. One possibility is that because people are especially honest about socially sensitive issues when taking online tests anonymously, our data are consistent with the view expressed by Sigmund Freud and others that both SS and OS attractions are experienced by most humans throughout their lives. Finally, following our description of a new empirical study, we offer a formal, predictive theory of sexual orientation – social pressure theory (SPT) – in both mathematical and computational formats. The theory explains how heteronormative pressure, which has varied throughout human history and which still varies from culture to culture, impacts the natural bisexual inclinations experienced by a substantial proportion of the population to produce what at first glance appear to be nonoverlapping categories of sexual orientation.

## Methods

The study is based on an analysis of scores obtained between January 1st, 2013 and October 6th, 2021 from a sample of 1,150,938 people in 215 countries and territories (63.9% from the US, UK, and Canada) on the English version of a validated online questionnaire of SO called the Epstein Sexual Orientation Inventory (ESOI), which was first posted online in 2006 (Epstein et al., 2012). The initial dataset contained 1,317,081 records and was cleaned as follows: records were deleted in which fewer than half the questions were answered or in which people reported English fluency under 6 on a scale from 1 to 10. For people who completed the questionnaire more than once on the same day, only the first record was retained.

The study was approved by the sponsoring institution’s federally registered Institutional Review Board as exempt research that involved minimal risk and that preserved the anonymity of participants. The study also qualified for a waiver of informed consent, in part to preserve the anonymity of participants [see HHS Federal Regulations 45 CFR 46.101(b)(4), 45 CFR 46.116(d), 45 CFR 46.117(c)(2), and 45 CFR 46.111].

Above the survey itself, people were asked general demographic questions. Participants’ ages ranged from 12 to 95 with a median of 18.0 (*M* = 21.7, *SD* = 10.1). The sample was 54.2% female, 43.0% male, and 2.7% other, with a racial/ethnic distribution as follows: 0.8% American Indian, 10.4% Asian, 5.6% Black, 7.3% Hispanic, 7.4% Other, and 68.4% White. Participants labeled their SO as follows: 1.2% asexual, 13.9% bisexual, 7.4% gay or lesbian, 2.8% other, 24.4% straight, and 50.3% unsure. Participants reported their educational attainment level as follows: 21.0% had not completed high school, 44.4% had a high school diploma, 4.0% had an associate’s degree or equivalent, 22.9% had a bachelor’s degree, 6.0% had a master’s degree, and 1.7% had a doctorate.

Participants were also asked whether they had ever changed their SO and to estimate, on two 10-point scales, how much uncertainty and distress they felt regarding their SO. The questionnaire itself contained 18 items, half of which asked about SS sexual fantasies, attractions, and behaviors, both past and present, and the other half of which asked about OS inclinations of the same sort (see Supplementary materials for the complete list of questionnaire items and the scoring method). Because compelling evidence that would allow us to weight the questionnaire items is so far lacking, we chose to weight each question equally.

Because the questionnaire was posted at a public website^1^, we had no control over demographic characteristics of the people who completed it. Upon completing the survey, people were given measures of their same-sex inclinations, opposite-sex inclinations, SOR (the range of points between and including the points associated with their SS and OS inclinations), MSO (the point half-way between the points associated with their SS and OS inclinations), and sex drive, all derived from questionnaire responses (see Supplementary Figure S1; Supplementary materials).

## Results

**Figure 1:**
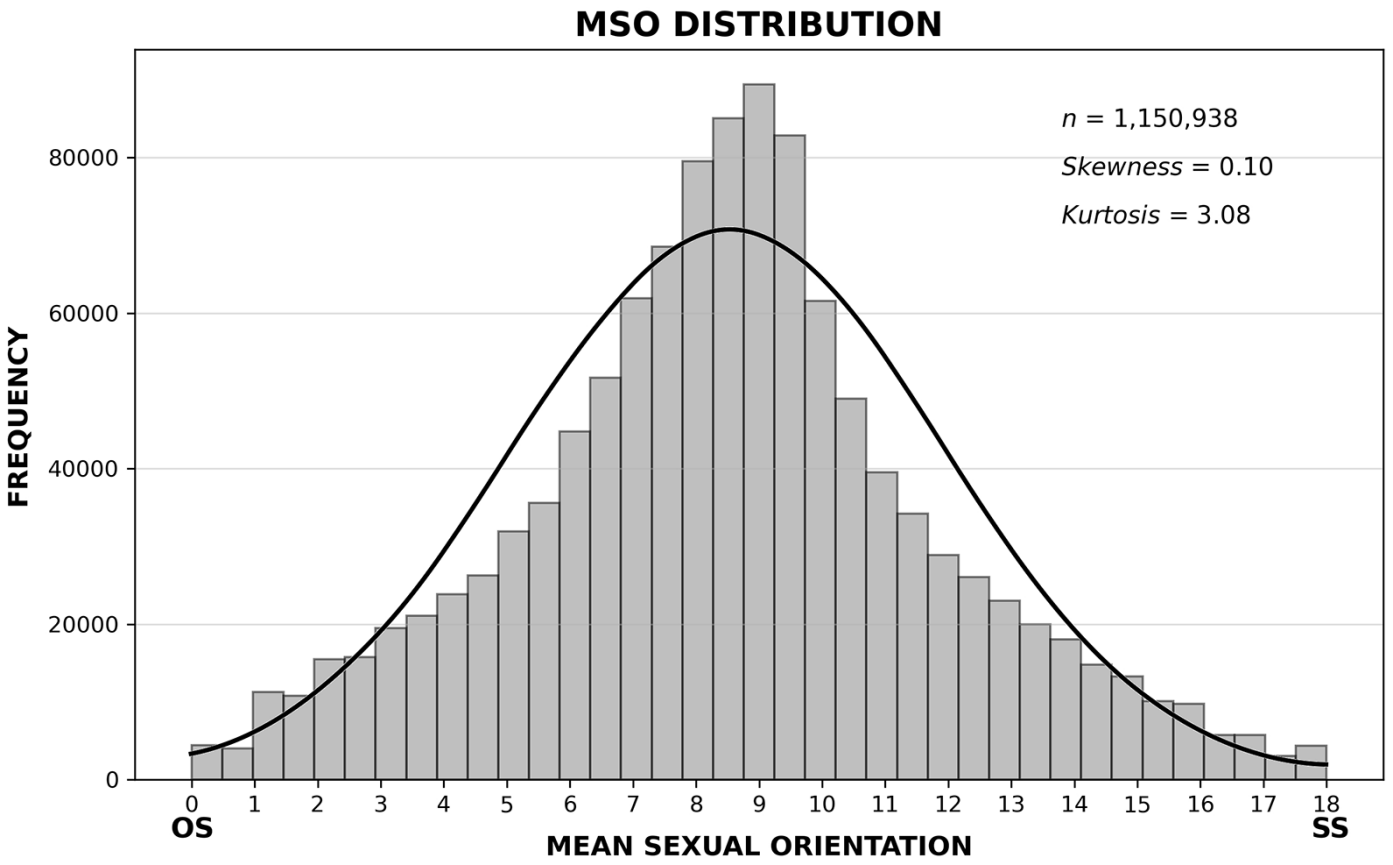
A histogram of mean sexual orientation (MSO) scores for the full dataset. The distribution is roughly normal in shape, and it is symmetrical about the modal questionnaire score of 9. For a normal curve, skewness would equal 0 (rather than 0.10), and kurtosis would equal 3 (rather than 3.08).

**Figure 2:**
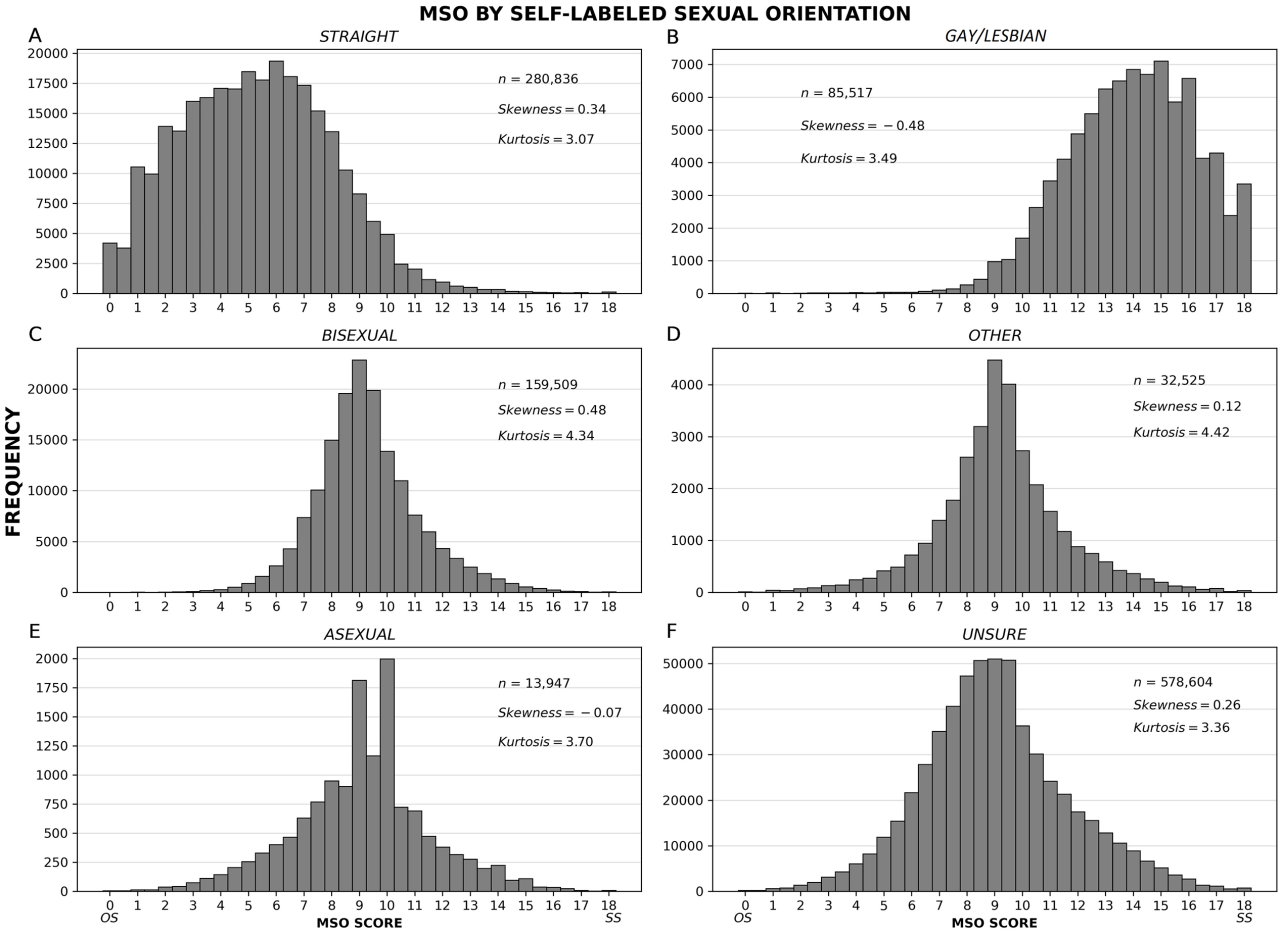
Frequency distributions of MSO scores by self-labeled sexual orientation. Separate distributions are shown for people self-labeled as follows: straight, gay/lesbian, bisexual, other, asexual, and unsure **(A–F)**. If discrete categories were sufficient to define SO, we would see non-overlapping distributions. Instead, each SO label corresponds to a broad, skewed distribution, meaning (a) that the labels do not describe discrete categories, and (b) that there is often a mismatch between the label people use to describe their SO and their actual sexual attractions, fantasies, and behaviors.

A histogram of MSO scores for the full dataset is roughly normal in shape (Figure 1) with a small positive skew, which means, roughly, that the distribution leans slightly toward the OS end of the SO continuum. The distribution is also unimodal, and the mode is at the center of the continuum (*M* = 8.5, *SD* = 3.4, mode = 9.0, median = 8.5). Popular categorical conceptions of SO imply that this graph should show a large spike at the OS end of the continuum and, perhaps, a small spike at the SS end, but that is not what we found. SO self-labels corresponded to broad, skewed distributions of MSO scores, which ranged across the entire SO continuum for each label (Figure 2).

**Figure 3:**
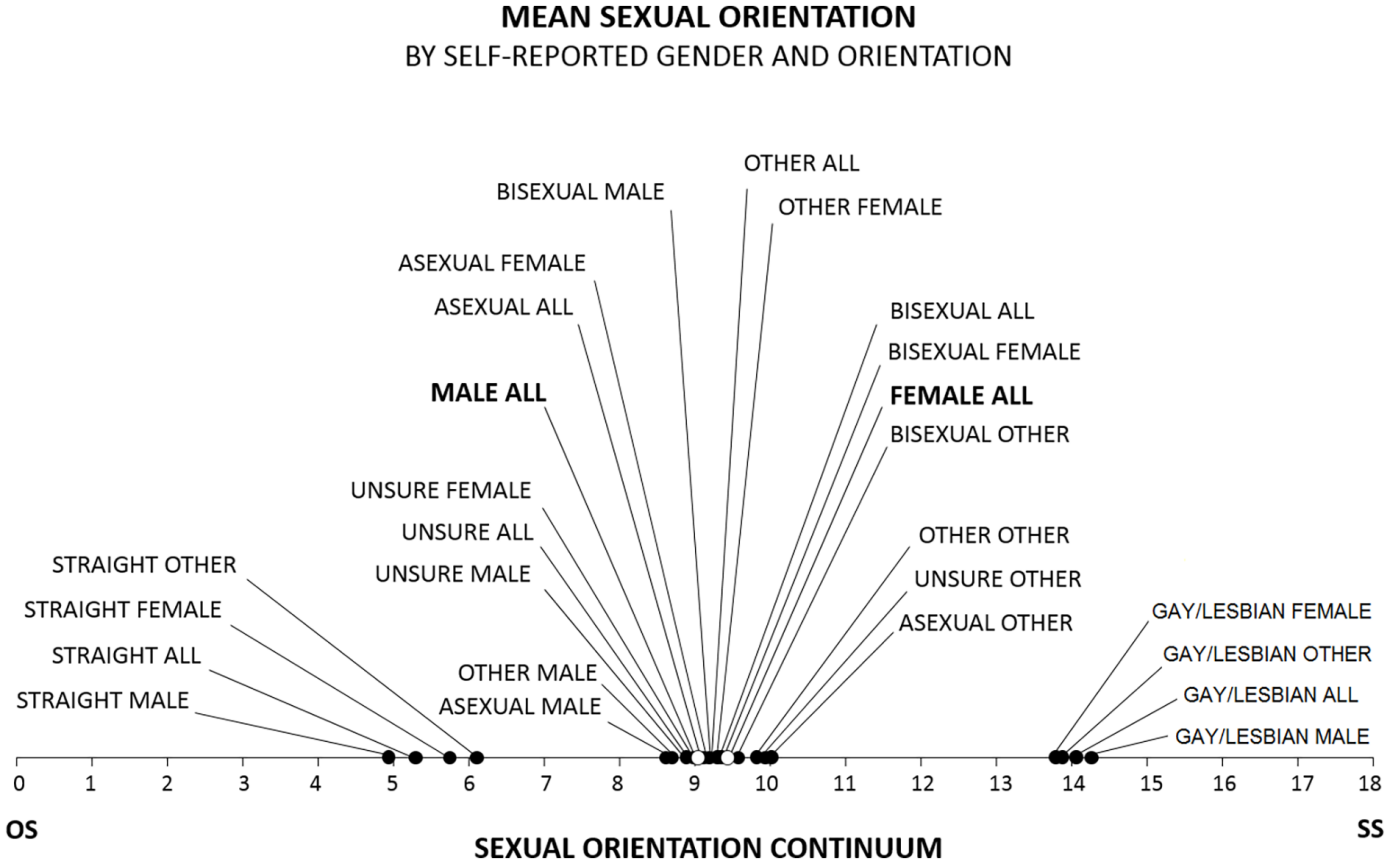
Mean MSO scores for self-labeled gender and orientation. Consistent with the generally understood meanings of the labels, MSO scores for self-labeled gays/lesbians clustered toward the SS end of the SO continuum; scores for self-labeled asexuals, self-labeled bisexuals, self-labeled others, and self-labeled unsures clustered toward the center; and scores for self-labeled straights clustered toward the OS end. In addition, the mean MSO score for females was significantly higher than the mean MSO score for males.

MSO scores distinguished people by gender in ways that are consistent with other research findings (Ellis et al., 1987; Haslam, 1997; Sell, 1997; Garnets and Peplau, 2000; Kraemer et al., 2006; Diamond, 2008; Thompson and Morgan, 2008; Chivers et al., 2010; Epstein et al., 2012; Ross et al., 2012; Vrangalova and Savin-Williams, 2012; Epstein and Robertson, 2014): self-labeled females scored more toward the SS end of the continuum than self-labeled males, for example (*M*_Male_ = 8.2, *SD* = 3.8; *M*_Female_ = 8.7, *SD* = 3.0; *M*_Other_ = 10.0, *SD* = 2.7; Kruskal-Wallis *H* = 13,434.46, *p* < 0.001) (Figure 3). MSO scores also distinguished people by SO self-label (*M*_Straight_ = 5.3, *SD* = 2.7; *M*_Unsure_ = 9.0, *SD* = 2.7; *M*_Asexual_ = 9.1, *SD* = 2.5; *M*_Other_ = 9.2, *SD* = 2.3; *M*_Bisexual_ = 9.3, *SD* = 1.9; *M*_Gay/Lesbian_ = 13.9, *SD* = 2.3; *H* = 454,072, *p* < 0.001) (Figure 3). Note: because ESOI scores are on an ordinal scale, we employ nonparametric statistical tests, such as the Kruskal-Wallis *H* test, Spearman’s rho (*ρ*), and the Mann–Whitney *U* test, throughout this report. Means and standard deviations are reported for comparison purposes, although the appropriateness of their use with ordinal data has long been debated (e.g., Lord, 1953; Townsend and Ashby, 1984).

MSO scores were substantially higher for people who said their SO had changed than for people who said their SO had never changed (*M*_Changed_ = 9.6, *SD* = 2.7; *M*_Unchanged_ = 7.5, *SD* = 3.7; Mann-Whitney *U* = 2.3 × 10^11^, *p* < 0.001; Cohen’s *d* = 0.65). MSO also differed significantly across racial and ethnic groups (*M*_AmericanIndian_ = 8.6, *SD* = 3.4; *M*_Asian_ = 8.2, *SD* = 3.5; *M*_Black_ = 8.3, *SD* = 3.4; *M*_Hispanic_ = 8.5, *SD* = 3.4; *M*_Other_ = 8.5, *SD* = 3.3; *M*_White_ = 8.6, *SD* = 3.4; *H* = 1,186, *p* < 0.001), and by educational attainment level (*M*_None_ = 9.0, *SD* = 3.4; *M*_HighSchool_ = 8.5, *SD* = 3.4; *M*_Associate_ = 8.4, *SD* = 3.2; *M*_Bachelors_ = 8.3, *SD* = 3.3; *M*_Masters_ = 8.2, *SD* = 3.5; *M*_Doctorate_ = 8.4, *SD* = 3.7; *H* = 7,943, *p* < 0.001). MSO scores also differed significantly between participants in the US, UK, and Canada combined and participants in other countries (*U* = 4.0 × 10^11^, *p* < 0.001), but both the effect size and the absolute difference between the mean scores were small (*M*_UsUkCan_ = 8.6, *SD* = 3.4; *M*_OtherCountries_ = 8.5, *SD* = 3.4; *d* = 0.03) (Supplementary Figure S2).

**Figure 4:**
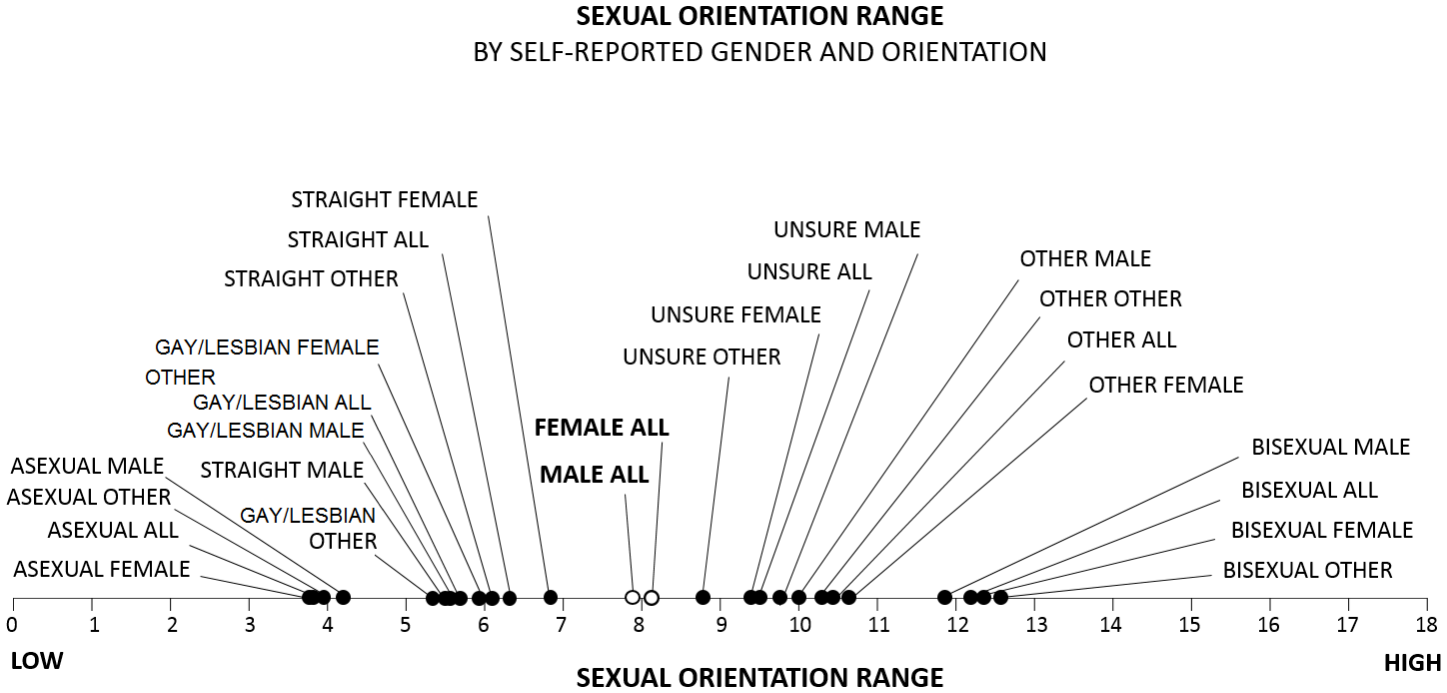
Mean sexual orientation range (SOR) scores for self-labeled gender and orientation. SOR scores clustered for different groups. Self-labeled bisexuals had the greatest range; self-labeled others had a smaller range; and self-labeled gays/lesbians and straights had even smaller ranges, with self-labeled asexuals having the smallest range.

Like MSO scores, SOR scores fell along the entire range of possible scores (0–18), with a mean of 9.4 (*SD* = 4.5) and, consistent with the findings of other researchers (Festinger, 1957; Festinger and Carlsmith, 1959; Elliot and Devine, 1994; Chung and Katayama, 1996; Sell, 1997; Baumeister, 2000; Garnets and Peplau, 2000; Peplau, 2001; Russell and Seif, 2001; Weinrich and Klein, 2002; Cooper, 2007; Diamond, 2008; Chivers et al., 2010; Epstein et al., 2012; Lippa, 2012; Mock and Eibach, 2012; Ross et al., 2012; Vrangalova and Savin-Williams, 2012; Epstein and Robertson, 2014), differed significantly by gender (*M*_Female_ = 9.7, *SD* = 4.3; *M*_Male_ = 9.0, *SD* = 4.8; *M*_Other_ = 9.3; *SD* = 4.8; *H* = 3,875, *p* < 0.001; *d* = 0.16). Self-labeled females also scored higher than self-labeled males in a pairwise comparison (*U* = 1.5 × 10^11^, *p* < 0.001, *d* = 0.15). Also consistent with other research (Sell, 1997; Weinrich and Klein, 2002; Rosario et al., 2006; Mock and Eibach, 2012), SOR scores for self-labeled bisexuals were substantially higher than SOR scores for self-labeled straights and gays/lesbians combined (*M*_Bisexual_ = 12.7, *SD* = 3.2; *M*_GayLesbianStraight_ = 6.1, *SD* = 4.3; *U* = 1.2 × 10^10^, *p* < 0.001; *d* = 1.74) (Figure 4). SOR scores also differed significantly by racial or ethnic group (*M*_AmericanIndian_ = 9.2, *SD* = 4.7; *M*_Asian_ = 8.1, *SD* = 4.4; *M*_Black_ = 9.4, *SD* = 4.5; *M*_Hispanic_ = 9.4, *SD* = 4.5; *M*_Other_ = 9.3, *SD* = 4.5; *M*_White_ = 9.6, *SD* = 4.6; *H* = 12,822, *p* < 0.001) and educational attainment level (*M*_None_ = 8.3, *SD* = 4.4; *M*_HighSchool_ = 9.3, *SD* = 4.5; *M*_Associates_ = 10.6, *SD* = 4.5; *M*_Bachelors_ = 10.1, *SD* = 4.5; *M*_Masters_ = 10.3, *SD* = 4.6; *M*_Doctorate_ = 10.0, *SD* = 4.9; *H* = 30,212, *p* < 0.001).

**Figure 5:**
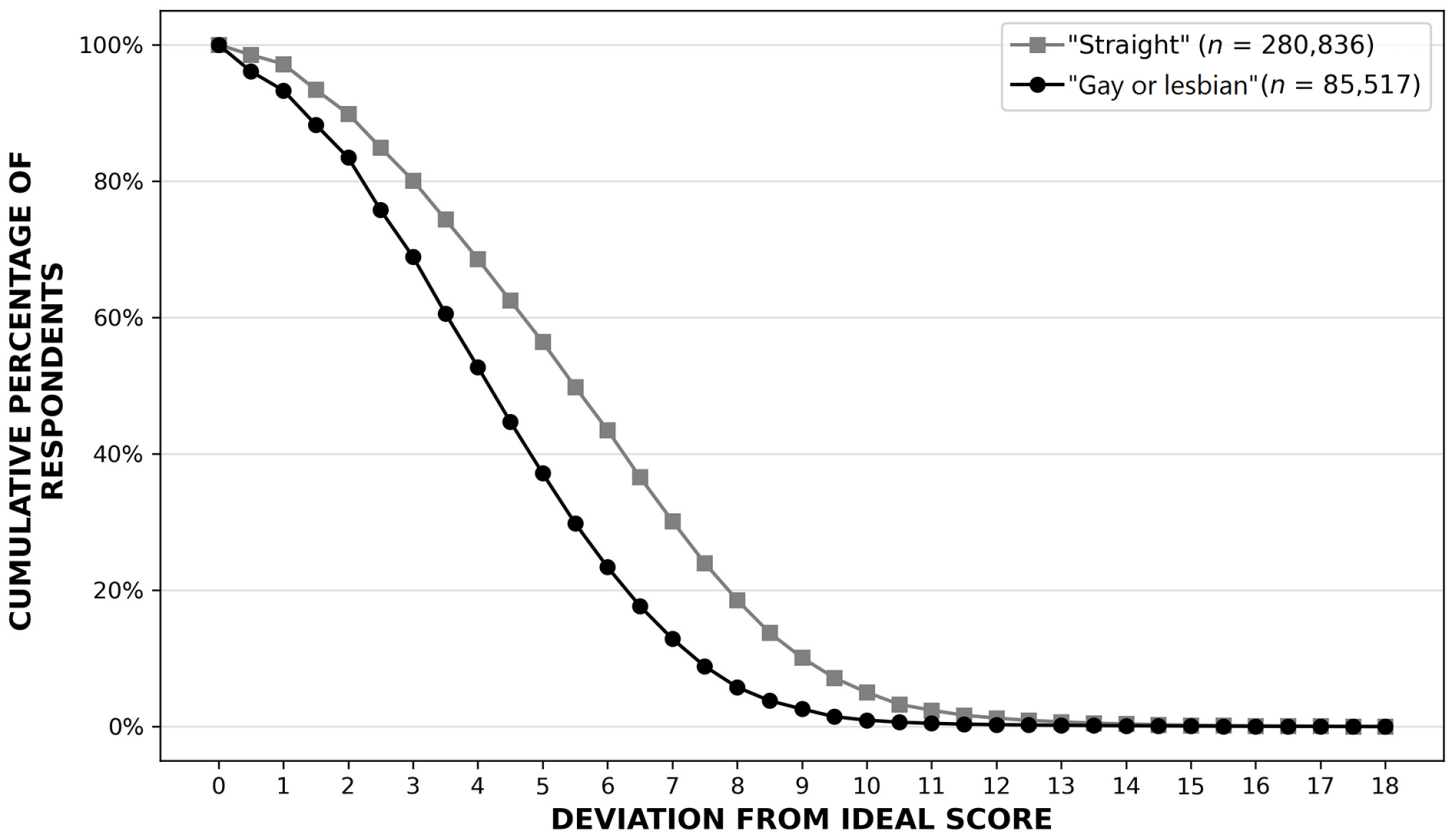
MSO deviation scores for self-labeled gays/lesbians and straights. The patterns are similar for each group, but the deviation levels are higher for self-labeled straights.

The mismatch between SO labels and MSO scores can be represented by deviation curves as shown in Figure 5. These curves show the cumulative percentages of people self-labeled gay/lesbian and straight who deviated from their expected MSO scores (0 for self-labeled straights, 18 for self-labeled gays/lesbians) by a range of distances on the SO continuum. The deviation patterns for self-labeled gays/lesbians and straights were similar, but deviation levels were higher for self-labeled straights. Overall, about 60% of our participants deviated from their expected MSO scores by four points or more on the 19-point continuum, about 22% by seven points or more, and about 6% by nine points or more.

As one might expect, we found a positive relationship between the level of uncertainty people reported regarding their SO and the level of distress they reported regarding their SO (Spearman’s *ρ =* 0.63, *p* < 0.001). At first glance, the relationship between the level of distress people reported regarding their SO and their deviation scores appeared to be relatively weak (*ρ* = 0.14, *p* < 0.001). A different picture emerged, however, when we broke down this relationship demographically. For self-labeled gays/lesbians, the relationship between the distress they reported and their deviation scores was relatively weak (*ρ* = 0.10, *p* < 0.001). For self-labeled *straights*, however, this relationship was stronger (*ρ* = 0.29, *p* < 0.001) and it was stronger still for self-labeled straight males (*ρ* = 0.36, *p* < 0.001; straight females: *ρ* = 0.23, *p* < 0.001).

## Discussion

Research on cognitive dissonance is relevant here. In most societies, people who call themselves bisexual, gay, or lesbian have already resolved a serious dissonance problem – that is, the mismatch between one’s socially prescribed SO label (“straight”) – and one’s actual sexual inclinations. By coming out, they have adopted a label that is more consistent with their sexual inclinations, shifting away from a discrepancy that caused them discomfort to a new kind of discrepancy which, by comparison, causes them less discomfort (Elliot and Devine, 1994; Schick et al., 2012). Looking at the issue in terms of Festinger’s original formulation (Festinger, 1957), self-labeled straights experience dissonance when they stray from opposite-sex inclinations because they cannot easily attribute their behavior to external factors. Mainstream society is telling them to be straight, so when they deviate from the societal norm, they will tend to blame themselves (Hinrichs and Rosenberg, 2002; Dodge et al., 2016; Bettinsoli et al., 2019; Mendos et al., 2020). When external factors are strong, discrepant behavior can easily be rationalized and dissonance is low, but when external factors are weak, dissonance is high (Festinger, 1957; Festinger and Carlsmith, 1959; Cooper, 2007).

Because males are generally allowed less flexibility in SO than females are (Hinrichs and Rosenberg, 2002; Dodge et al., 2016; Bettinsoli et al., 2019), Festinger’s theory predicts greater dissonance for males than females, which is consistent with our findings. The issue can also be viewed from a self-concept perspective. Because SO is an integral part of one’s self-concept, self-labeled straights who stray from their prescribed sexual inclinations are in conflict with both their sexual identity (a part of the self-concept) and societal norms, whereas self-labeled gays/lesbians who stray may be in conflict only with their sexual identity (Larson, 1981; Thibodeau and Aronson, 1992).

Through much of his career, Sigmund Freud insisted that bisexuality was the natural state for human beings. In the 1910 edition of his book, *Three Contributions to the Theory of Sex*, he spoke of “the original predisposition to bisexuality,” adding that “without taking into account the factor of bisexuality it will hardly be possible to understand the actually observed sexual manifestations in man and woman” (Freud, 1910). He went further in the 1920 edition, insisting that “psychoanalytic investigation very strongly opposes the attempt to separate homosexuals from other persons as a group of a special nature” (Freud, 1920). To Freud, heterosexuality and homosexuality were unnatural states resulting from the suppression of natural bisexual tendencies (Freud, 1910, 1937). Although Freud sometimes used the term “bisexual” to refer to individuals possessing both male and female anatomy (Freud, 1910), at other times he defined it as the ability to “take as [one’s] sexual objects persons of either sex without the one trend interfering with the other,” later declaring that “all human beings are bisexual in this sense and their libido is distributed between objects of both sexes, either in a manifest or a latent form” (Freud, 1937). He also developed this theme in his classic *Civilization and Its Discontents* (Freud, 1930) and other works (e.g., Freud, 1950). Kinsey’s views on bisexuality overlapped with Freud’s (Young-Bruehl, 2001), and so did the views of sociobiologist E. O. Wilson, who noted that bisexual behavior is common among mammals and also spoke of the “true bisexuality latent within the brain” (Wilson, 1978).

Occasional or frequent same-sex sexual behavior has been observed in more than 1,500 animal species (Bagemihl, 1999; Terry, 2000; Roughgarden, 2004; Bailey and Zuk, 2009; Scharf and Martin, 2013), including in chimpanzees, with whom humans share 98.7% of their genes (Prüfer et al., 2012). Bonobos, close relatives of the chimpanzee with whom we also share 98.7% of our genes (Prüfer et al., 2012), are virtually all bisexual and far less violent than chimpanzees (Savage-Rumbaugh and Wilkerson, 1978; Hashimoto, 1997; Bagemihl, 1999; Fruth and Hohmann, 2006). The ubiquity of same-sex sexual behavior in the animal kingdom is not enough to prove that bisexuality is the human norm, but it does point to the possibility that engaging in both SS and OS sexual behaviors was an ancestral condition possessed by some of the earliest sexually-reproducing organisms (Monk et al., 2019).

In any case – and setting aside for the moment the limitations of our sample – although our data could be said to be consistent with Freud’s claim that bisexuality is the human norm, we do not make that claim. A more conservative interpretation of our findings is that in the absence of social pressure to be straight, most people would experience a mix of SS and OS inclinations at various times in their life. On a histogram of sexual orientation scores of the sort we present in Figure 1, such inclinations would be normally distributed. Where heteronormativity reigns, one might expect to see that among people who avoid labeling themselves straight, bisexuality would be common, and research tends to support that assertion (Gates, 2011; Bailey et al., 2016; Ipsos, 2021; Jones, 2023). But here things get murky. As Yoshino (2000) pointed out, and as others have reiterated (Eliason, 2000; Bowes-Catton, 2021), monosexuals tend to feel threatened by the existence of bisexuals, and this has led to a virtual erasure of the bisexual label. We live, it seems, in a world in which strong social pressure exists for people to call themselves straight, and that same world also puts pressure on nonheterosexuals to call themselves gay, even though many people in both camps experience both SS and OS tendencies. In a sense, the strongest social pressure of all is for people not to be bisexual! If our theory is correct, that pressure is almost outrageously ironic.

### Social pressure theory (SPT) of sexual orientation

Our data are consistent with a theory of sexual orientation that can be stated as follows: same-sex (SS) and opposite-sex (OS) inclinations in human beings (a) are independent of each other, (b) coexist in individuals in different proportions, and (c) will be roughly normally distributed in a population in which no social pressure exists to push people toward SS or OS inclinations. When net social pressure favors one inclination (say, OS inclinations), the normal distribution becomes skewed as it drives people toward that inclination. At some point, the normal curve (which is comprised of two separate curves, one for each inclination) appears to break, resulting in a bimodal distribution in which a large mode exists at one end of the distribution (OS, in modern society) and a smaller mode at the other (SS). This creates the impression that two types of sexual orientation exist – or even, to some, the impression that two types of *people* exist – but the second mode is simply an artifact of social pressure. It consists of people who resist social pressure to change their sexual inclinations, no matter how strong that pressure.

**Figure 6:**
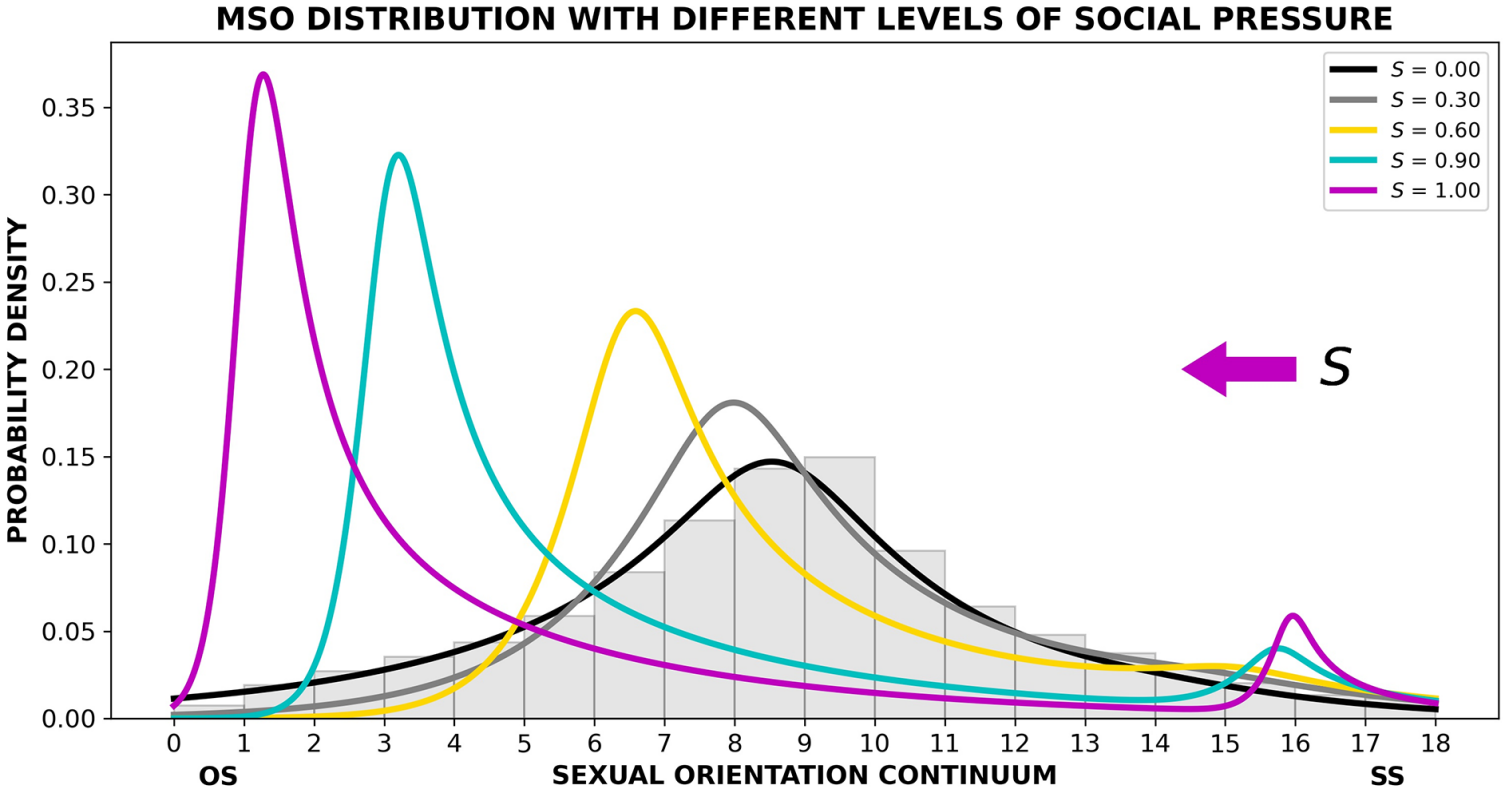
Probability density curves generated by the social pressure theory (SPT) model showing the impact of different levels of social pressure (*S*). The gray bars represent the new data collected in the present study. The black curve is generated by the SPT model when *S* = 0. As social pressure increases (gray curve, then yellow, then cyan, then magenta), a second mode appears at the SS end of the SO continuum.

A mathematical representation of this theory is delineated in Supplementary materials. This model contains no free parameters; all parameters were calculated from the dataset we have presented in this study. According to SPT, the MSO distribution results from a linear combination of two sinh-arcsinh distributions (Kokoska and Zwillinger, 2000; Jones and Pewsey, 2009) at certain mixture rates; the separate distributions represent independent SS and non-SS sexual inclinations. Figure 6 shows how the MSO distribution becomes increasingly distorted as social pressure (*S*) increases (also see Supplementary Figure S3). Although social pressure in the modern world almost universally pushes the curve toward the OS end of the continuum, the model suggests that pressure toward the SS end of the continuum will produce symmetrical changes in that direction. The vision of a world in which only SS inclinations are acceptable – in some instances as a means of limiting population growth – has been explored over the last 50 years in a number of short stories, books, and movies (e.g., Haldeman, 1974; Grugman, 2008; Beier, 2012; Preble, 2013; Budd, 2016; Love is All You Need, 2016). The predictive and symmetrical nature of the theory can be explored using a computational implementation of the SPT equations, written in Python.^2^

The present study is one of several that support three important findings about SO, namely that (a) SO lies on a continuum (Ellis et al., 1987; Haslam, 1997; Sell, 1997; Kraemer et al., 2006; Thompson and Morgan, 2008; Chivers et al., 2010; Epstein et al., 2012; Vrangalova and Savin-Williams, 2012; Epstein and Robertson, 2014; Savin-Williams, 2014; *cf.* Gangestad et al., 2000; Savin-Williams and Vrangalova, 2013; Norris et al., 2015), (b) SO labels correspond to broad, skewed, overlapping distributions of measures of sexual inclinations (Ellis et al., 1987; Haslam, 1997; Sell, 1997; Kraemer et al., 2006; Thompson and Morgan, 2008; Chivers et al., 2010; Epstein et al., 2012; Vrangalova and Savin-Williams, 2012; Epstein and Robertson, 2014), and (c) SO expression is both flexible and fluid to some extent (Chung and Katayama, 1996; Garnets and Peplau, 2000; Rosario et al., 2006; Diamond, 2008; Ott et al., 2011; Ross et al., 2012; Savin-Williams et al., 2012; Savin-Williams and Vrangalova, 2013; Norris et al., 2015; Morandini et al., 2021; Xu et al., 2021; Srivastava et al., 2022a,b).

To these findings, the present study adds: (d) most humans experience a mixture of SS and OS tendencies throughout their lives (e) in the absence of social pressure, SS and OS sexual inclinations will likely be normally distributed on a continuum between extreme forms of those inclinations, (f) the label “homosexual” describes a second mode that appears in the distribution when society demands that people engage exclusively in OS sexual behavior, and (g) the greater the mismatch between people’s sexual orientation labels and their actual sexual inclinations, the more distress they feel about their sexual orientation.

### Limitations, strengths, and future research

The proportion of self-labeled non-straight individuals in our sample (75.6%) was well above the proportion generally believed to exist in the general population of the US and other countries – roughly 3 to 8% (Bostwick et al., 2010; Herbenick et al., 2010, 2017; Joloza et al., 2010; Ward et al., 2014; Copen et al., 2016). High non-straight participation in our study was, at least in part, an artifact of internet sampling; our questionnaire attracted a large number of people who were unsure about their sexual orientation. There is another possibility, however, that cannot be ruled out: namely, that because people could complete our questionnaire in complete anonymity, they were more honest about their sexual inclinations than they would be with more invasive surveys. A number of recent studies have shown that the more invasive the survey method, the more likely people are to lie about socially sensitive issues (Ong and Weiss, 2000; Trau et al., 2013; Robertson et al., 2017), answering questions mainly with socially acceptable answers; this effect has been shown to be especially robust for sexual orientation (Robertson et al., 2017). In other words, although sampling bias cannot be ruled out in the present study – thus limiting the generalizability of our findings – it is also possible that this study is bringing us closer to the truth about how sexual orientation varies in a population. Ample evidence suggests that estimates of the prevalence of non-heterosexuality obtained using conventional survey methods almost certainly underestimate such prevalence.

We cannot determine definitively to what extent the near-normal MSO distribution we found in this study was an artifact of internet sampling. We conducted four analyses, however, to try to shed some light on this issue:

1. We drew random samples from the US portion of our full sample that closely matched four demographic characteristics of the US population: age, race/ethnicity, gender, and educational attainment (see Supplementary Table S1). This sample did not yield a curve with a strong positive skew; the resulting MSO distribution was near normal in shape (Supplementary Figure S4, *Skp* = 0.11), closely resembling our original curve (Figure 1, *Skp* = 0.10).
2. We compared people who said they had changed their sexual orientation to people who said they had not. The former sample produced a somewhat positively skewed MSO distribution (Supplementary Figure S5A, *Skp* = 0.38); the latter produced a more positively skewed curve (Supplementary Figure S5B, *Skp* = 0.42).
3. We compared people who said they felt considerable distress about their sexual orientation (8 or over on a scale from 1 to 10) to people who said they felt little distress about their sexual orientation (under 4 on that scale). The former sample produced a near-normal MSO distribution (Supplementary Figure S6A, *Skp* = 0.06); the latter produced a somewhat positively skewed curve (Supplementary Figure S6B, *Skp* = 0.43).
4. We compared people who said they felt considerable uncertainty about their sexual orientation (8 or over on a scale from 1 to 10) to people who said they felt little uncertainty about their sexual orientation (under 4 on that scale). The former sample produced a near-normal MSO distribution (Supplementary Figure S7A, *Skp* = 0.14); the latter produced a somewhat positively skewed curve (Supplementary Figure S7B, *Skp* = 0.80). Combining people who experienced low distress and low uncertainty produced an MSO curve that was more positively skewed (Supplementary Figure S8A, *Skp* = 0.94), and combining those groups with people who said their sexual orientation had never changed increased the positive skew even further (Supplementary Figure S8B, *Skp* = 1.14).

This pattern of findings is informative, but it does not settle the sampling issue. It indicates that the near-normal curve we found is typical among people who are unsure about or distressed by their sexual orientation. This is also evident in the breakdown of the data shown earlier in Figure 2. People who are more certain or less distressed about their sexual orientation produce the positively skewed curves one might expect to see in heteronormative cultures (again, we see this pattern in Figure 2). But which distribution – if either – is natural for human beings? Our online questionnaire might be giving us an accurate look at the mix of people society creates when heteronormative pressure is high and people are given an opportunity to reveal their inclinations with complete anonymity, or our questionnaire might be attracting a disproportionately large number of people who are questioning their sexual orientation. The truth is probably somewhere in between, with both anonymity and sampling bias contributing to our findings. Note, however, that even when our distributions are skewed, MSO scores vary over a wide range – typically over the entire breadth of the SO continuum. None of our analyses supports either of the simplistic ideas about sexual orientation that dominate political discussions – namely, that everyone is naturally straight, or that most people are straight and some are gay (van der Toorn et al., 2020).

#### Analysis by age

**Figure 7:**
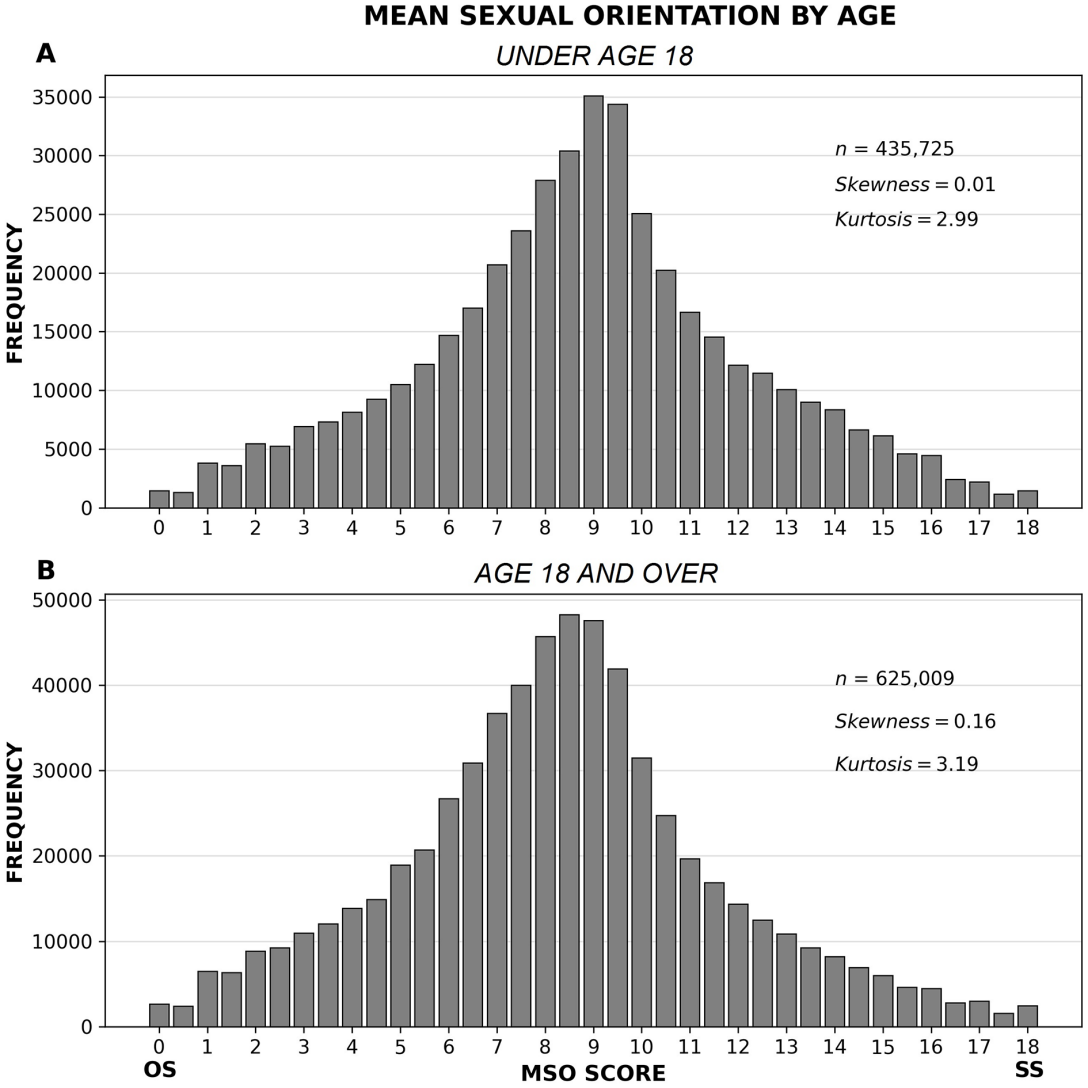
MSO by age. The MSO distributions for people under 18 **(A)** and people 18 and over **(B)** are similar in shape, with the distribution of scores for the younger group closer to normal.

As noted above, our sample also skewed toward young people (median age = 18.0). Since young people have less sexual experience, one might speculate that dividing the data by median age would yield two dramatically different MSO distributions – a near-normal curve for people under 18, and a strongly positively skewed curve for people 18 and over. Such a difference could also be considered a generational effect, with older people impacted by the stronger heteronormative pressure that typified the mid and late 20th century (Twenge et al., 2016; Rosati et al., 2020). That is not what we found, however. The MSO distributions of those age groups were similar in shape, with the curve for the younger group closer to normal (Figure 7) (Kolmogorov–Smirnov *D* = 0.09, *p* < 0.001). This still does not settle the sampling bias issue, but it is suggestive. The fact that the curve for the younger group is so close to normal (with both skewness and kurtosis differing from normal values by 0.01) is consistent with the possibility that most humans experience a mix of SS and OS tendencies throughout their lives, and that these tendencies are normally distributed in the population. Younger people have, generally speaking, been raised in a world in which heteronormative pressure has been declining (Levinson et al., 2020; Poushter and Kent, 2020), and they have also been subjected to such pressure for a shorter period of time than their elders have. Other large-scale surveys have found evidence that self-reported nonheterosexuality is higher in younger generations, with prevalence rates around 20% for Generation Z (of whom more than 60% are bisexual), versus about 12% for Millennials and 3% for Baby Boomers (Ipsos, 2021; Jones, 2023).

#### Analysis by country and region

More research is needed to shed light on how sexual orientation varies by culture and region (Salvati et al., 2020; Arcidiacono and Carbone, 2021; Salvati and Koc, 2022). Although our dataset is large enough to permit analyses by country and region, we have, mainly because of length considerations, elected to include such analyses in a separate paper that will summarize data from both the English version of the ESOI and translations of the questionnaire into Arabic, Chinese (simplified and traditional), French, German, Japanese, Spanish, and other languages. That paper will also show how the formal expression of SPT can be used to make specific predictions about the shape of SO distributions in different cultures. Of note in the present study is that the shape of the distribution of MSO scores for the US, the UK, and Canada combined (*n* = 735,076) is similar to the shape of the distribution of MSO scores for the other 212 countries and territories in the present study (*n* = 415,862) (Supplementary Figure S2).

#### Possible revisions

The binary nature of our scale limits its ability to fully represent the experience of non-binary individuals – a common criticism of continuum models of sexual orientation (Storms 1980; van Anders 2015; Galupo et al., 2017). Our questionnaire also does not explore “non-biological gender-related factors, partnered sexualities unrelated to gender or sex, or potential divergences between love and lust” (van Anders 2015). Some new scales, such as the Gender Inclusive Scale (Galupo et al., 2017) use more inclusive language than we employ in the ESOI, such as “other-gender,” rather than “opposite-sex.” We do not question the value of recent attempts to understand the multidimensional nature of sexual orientation and human intimacy. In part to introduce some degree of mathematical and predictive rigor into our understanding of sexual orientation, we limit our current investigation to the traditional binary scale, which is likely applicable at the moment to about 97% of humanity (Ipsos, 2021). The ESOI also yields scores for self-labeled asexuals that might seem odd at first glance. A self-labeled asexual will score 0 s for sex drive, OS inclinations, SS inclinations, and SOR, but that individual will score a 9 for MSO. We suggest that these five numbers taken together are meaningful to the self-labeled asexual, but someone might object to assigning that MSO of 9. Future versions of the ESOI will incorporate changing attitudes and language about gender and sexual orientation.

Unfortunately, ongoing societal debates about sexual orientation – often rancorous – are fueled by beliefs that are inconsistent with our findings – for example, the mistaken belief that categorical sexual orientation labels correspond to groups that are non-overlapping in their sexual inclinations (DeLamater and Hyde, 1998). Anti-gay sentiments, in particular, are often based on the faulty assumption that self-labeled straights have OS sexual inclinations exclusively; it would be difficult, presumably, to disapprove of homosexuality if one were aware of the wide range of sexual inclinations felt at times by many self-labeled straights. Educating the public about the scientific dimensions of sexual orientation might end or at least quell the debates, as well as give comfort to a large number of mislabeled people.

## Data availability statement

The raw data supporting the conclusions of this article will be made available by the authors, without undue reservation. The computational model is accessible at https://github.com/aibrt1/apps/blob/main/mso_simulation.py.

## Ethics statement

The studies involving human participants were reviewed and approved by the federally registered Institutional Review Board (IRB) of the sponsoring institution (American Institute for Behavioral Research and Technology). The IRB is registered with OHRP under number IRB00009303, and the Federalwide Assurance number for the IRB is FWA00021545. Written informed consent from the participants’ legal guardian/next of kin was not required to participate in this study in accordance with the national legislation and the institutional requirements.

## Author contributions

RE: conceptualization, methodology, investigation, supervision, writing—original draft, writing—reviewing, and editing. HW: statistical analysis, computational modeling, and visualization. VZ: statistical analysis, visualization, writing—reviewing, and editing. All authors contributed to the article and approved the submitted version.

## Funding

This work was supported by general funds of the American Institute for Behavioral Research and Technology, a nonpartisan, nonprofit, 501(c)(3) organization. This research did not receive any specific grant from funding agencies in the public, commercial, or not-for-profit sectors.

## Conflict of interest

The authors declare that the research was conducted in the absence of any commercial or financial relationships that could be construed as a potential conflict of interest.

## Publisher’s note

All claims expressed in this article are solely those of the authors and do not necessarily represent those of their affiliated organizations, or those of the publisher, the editors and the reviewers. Any product that may be evaluated in this article, or claim that may be made by its manufacturer, is not guaranteed or endorsed by the publisher.

## Footnotes

^1^ https://MySexualOrientation.com

^2^Accessible at https://github.com/aibrt1/apps/blob/main/mso_simulation.py.
