# Supplementary material for: Is everyone a mix of straight and gay? A social pressure theory of sexual orientation, with supporting data from a large global sample
Source: Front Psychol. 2023 Jul 11;14:1187377. doi: 10.3389/fpsyg.2023.1187377 (PMC10367109; doi:10.3389/fpsyg.2023.1187377)
Supplement: Supplementary file 1 [file Data_Sheet_1.pdf]

Supplementary Material

Is everyone a mix of straight and gay? A social pressure theory of sexual orientation, with supporting data from a large global sample

Robert Epstein, PhD*, Hongyu Wang, Vanessa R. Zankich

***Correspondence:** Corresponding Author: re@aibrt.org

Supplementary Figure S1. Screen capture of an example results page.

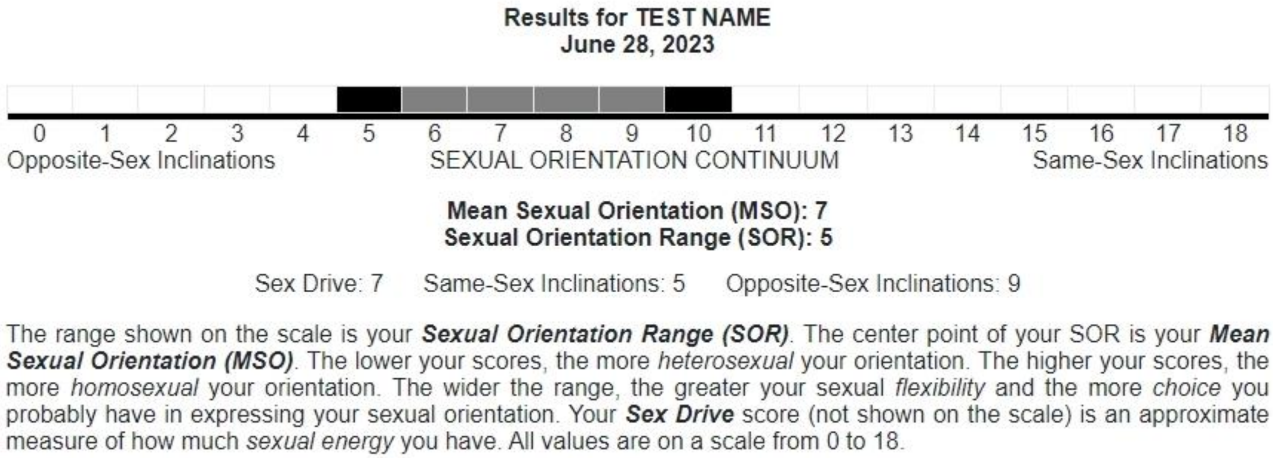

Note. This results page reflects what one might refer to as bisexuality.

Supplementary Figure S2. Mean sexual orientation (MSO) scores in the US, the UK, and Canada combined, vs. MSO scores in other countries.

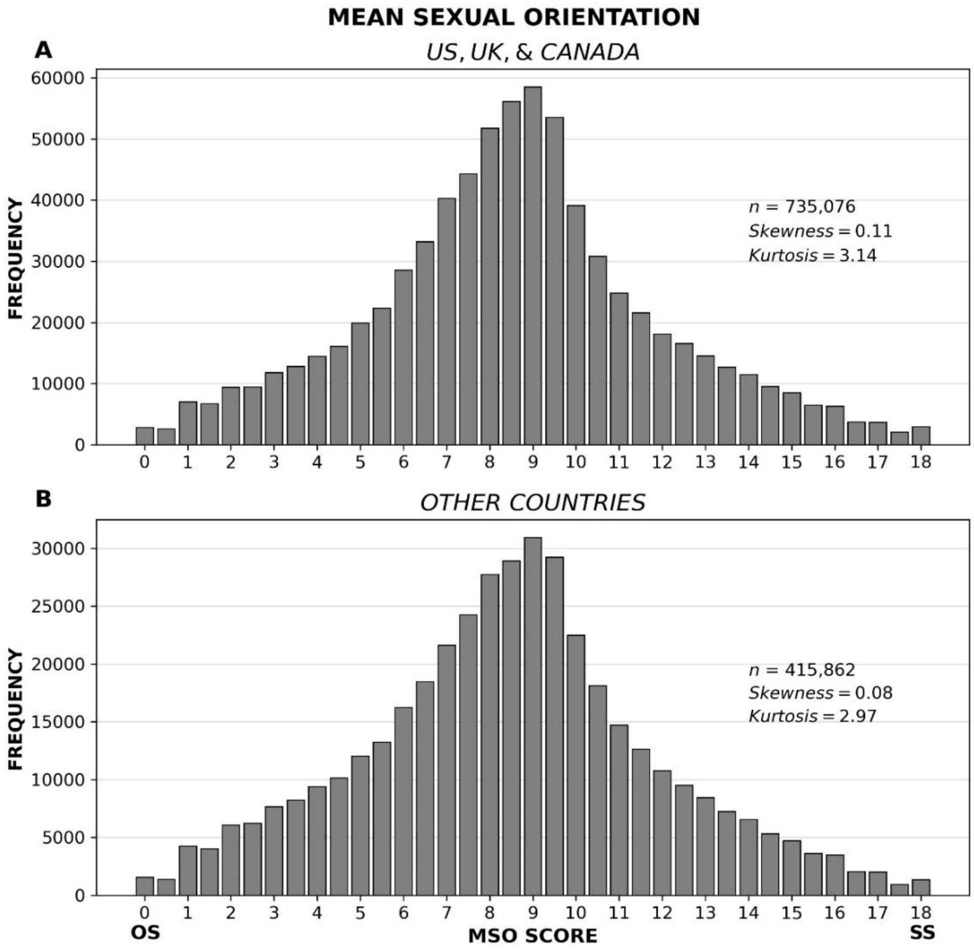

Note. Graph A shows a histogram of MSO scores in the US, the UK, and Canada combined. Graph B shows a histogram of MSO scores in the other 212 countries and territories in the present study. The shapes of the histograms are remarkably similar (but not, as it might appear at first glance, identical). An analysis of data by country and region will be presented by the authors in a separate paper, which will include data from the English version of the ESOI, along with data from the Arabic, Chinese (simplified and traditional), French, German, Japanese, Spanish, and other translations of the ESOI.

Supplementary Figure S3. MSO distribution by social pressure level (*S*).

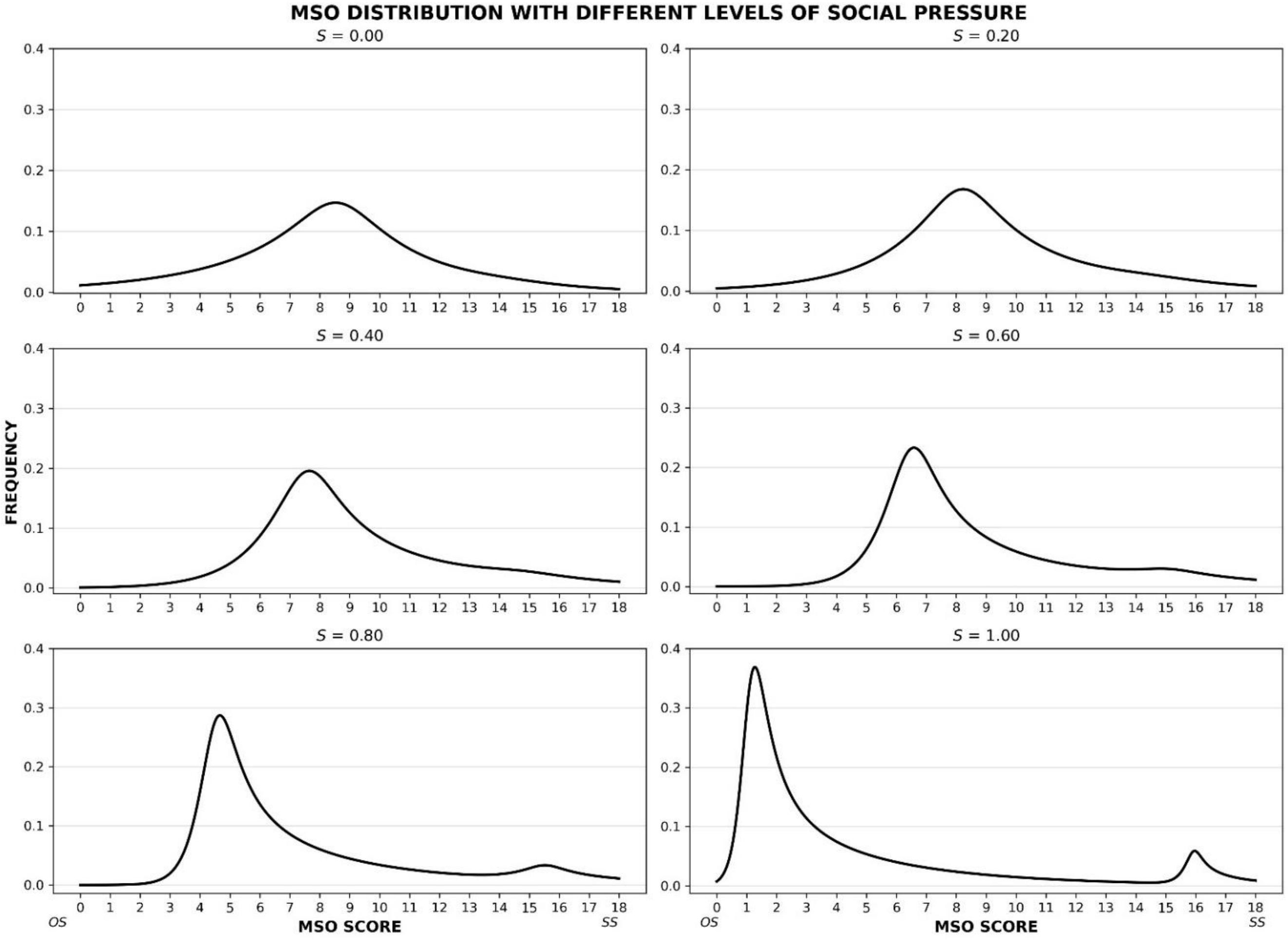

Note. The parameters used to create the above graphs are as follows, with only *S* changing in value:

p = 0.9257

(1-p) = 0.0743

μ_1 = 8.5222

μ_2 = 13.9438

τ_1 = 0.5995

τ_2 = 0.6000

σ_1 = 1.5366

σ_2 = 2.3170

υ_1 = -0.0965

υ_2 = -0.4762

Supplementary Figure S4. Sampling the dataset to match US demographic characteristics.

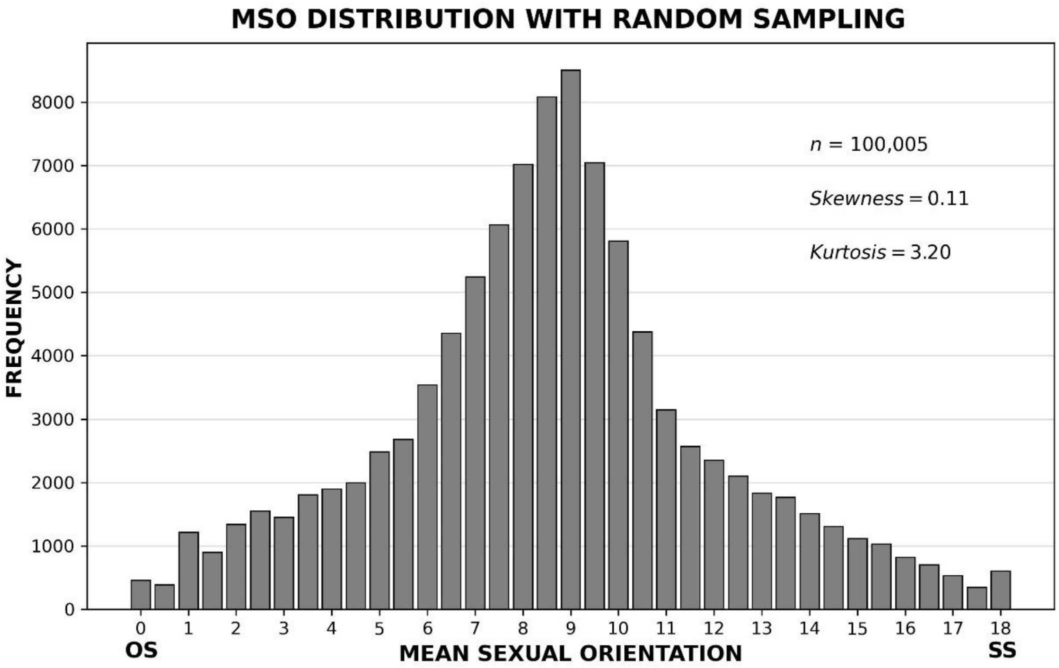

Note. The graph was created by taking random samples of individuals from the full dataset (n = 1,150,938) to match the proportions of age, race/ethnicity, gender, and educational attainment of the general population of the US as of 2019 (United States Census Bureau 2019a; United States Census Bureau 2019b). The resulting histogram is similar in shape to the histogram of the full dataset (Figure 1).

Supplementary Figure S5. Histograms of MSO scores for study participants who said they had changed (Graph A) and had never changed (Graph B) their sexual orientation.

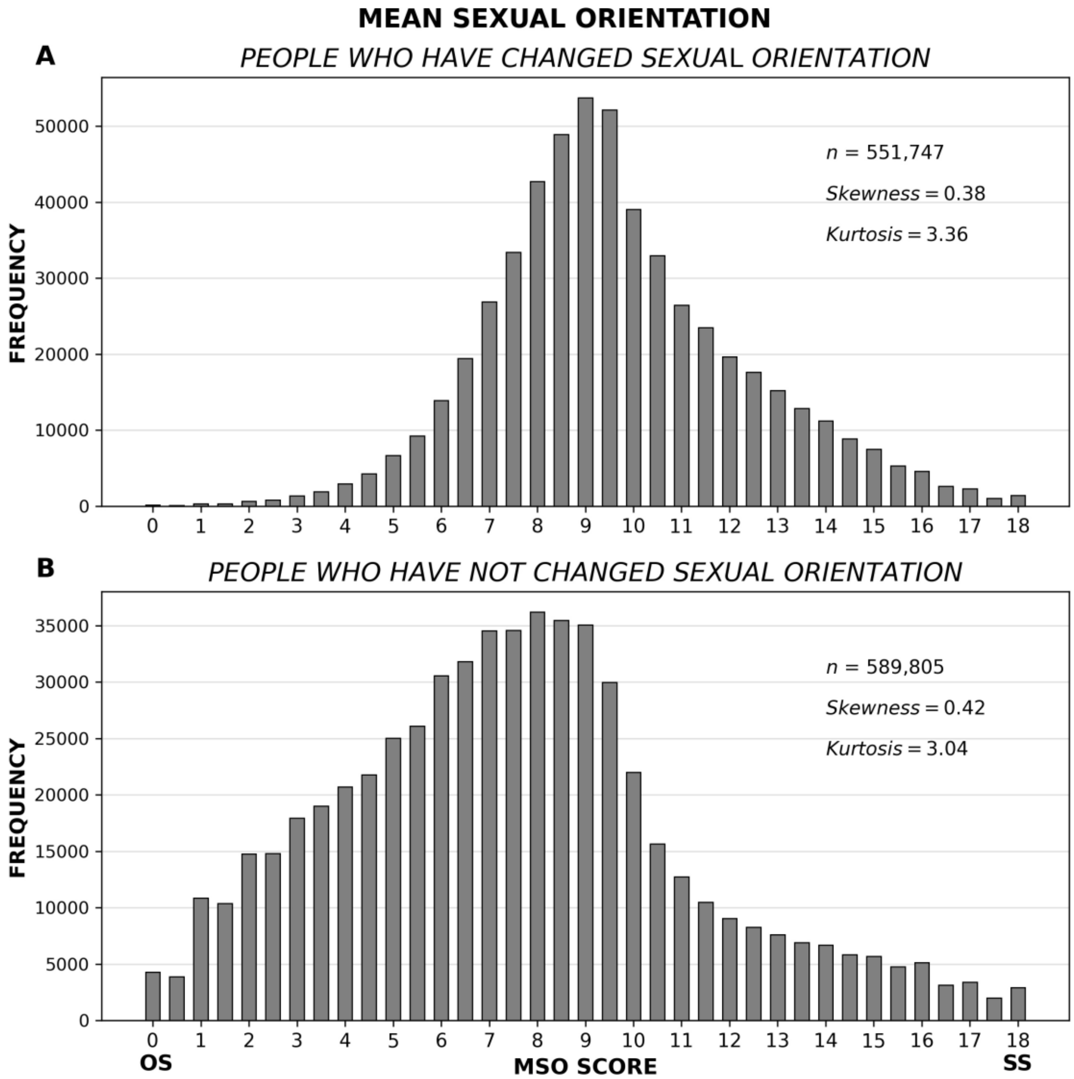

Supplementary Figure S6. Histograms of MSO scores for study participants reporting high distress (Graph A, values 8-10) and low distress (Graph B, values 1-3) regarding their sexual orientation.

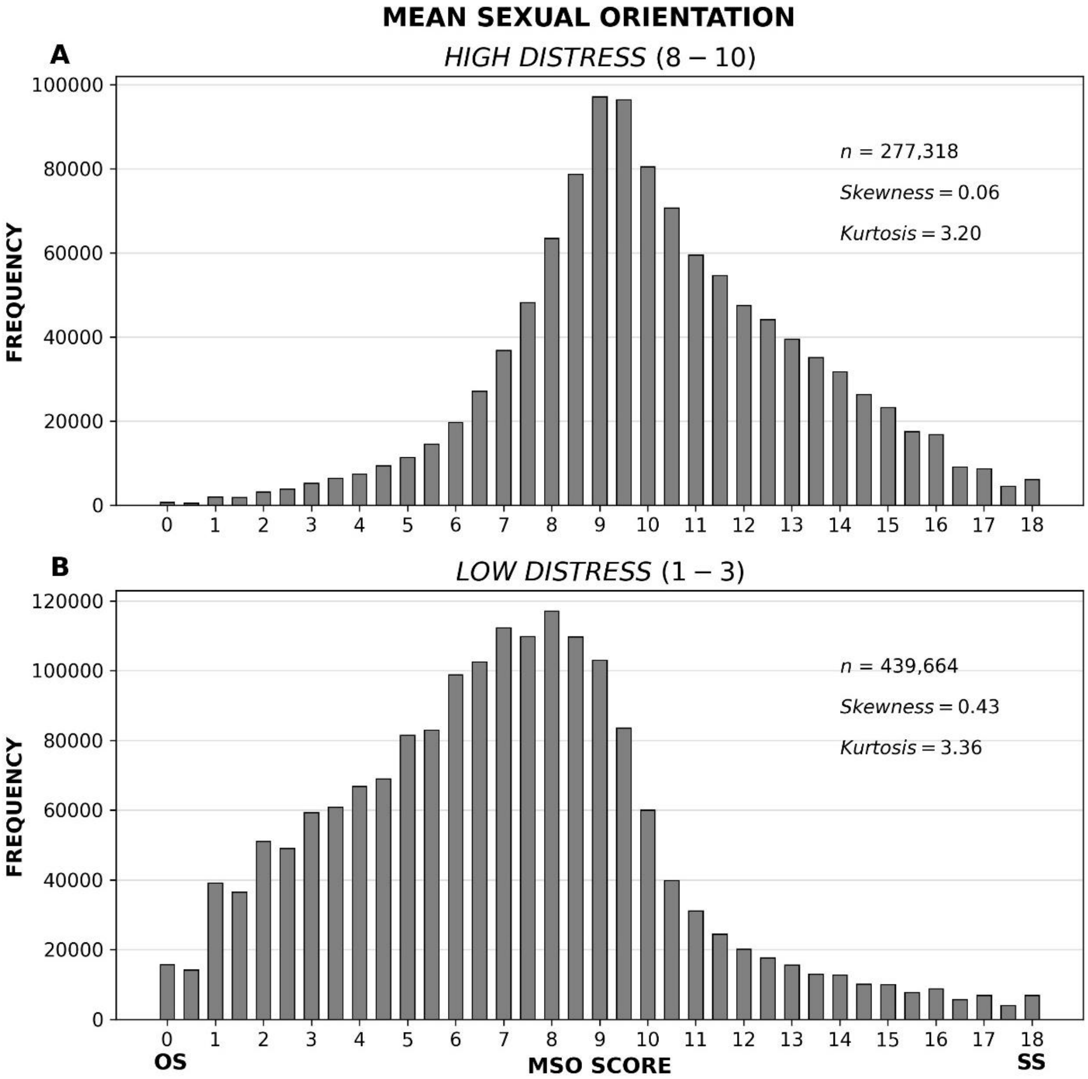

Supplementary Figure S7. Histograms of MSO scores for study participants reporting high uncertainty (Graph A, values 8-10) and low uncertainty (Graph B, values 1-3) regarding their sexual orientation.

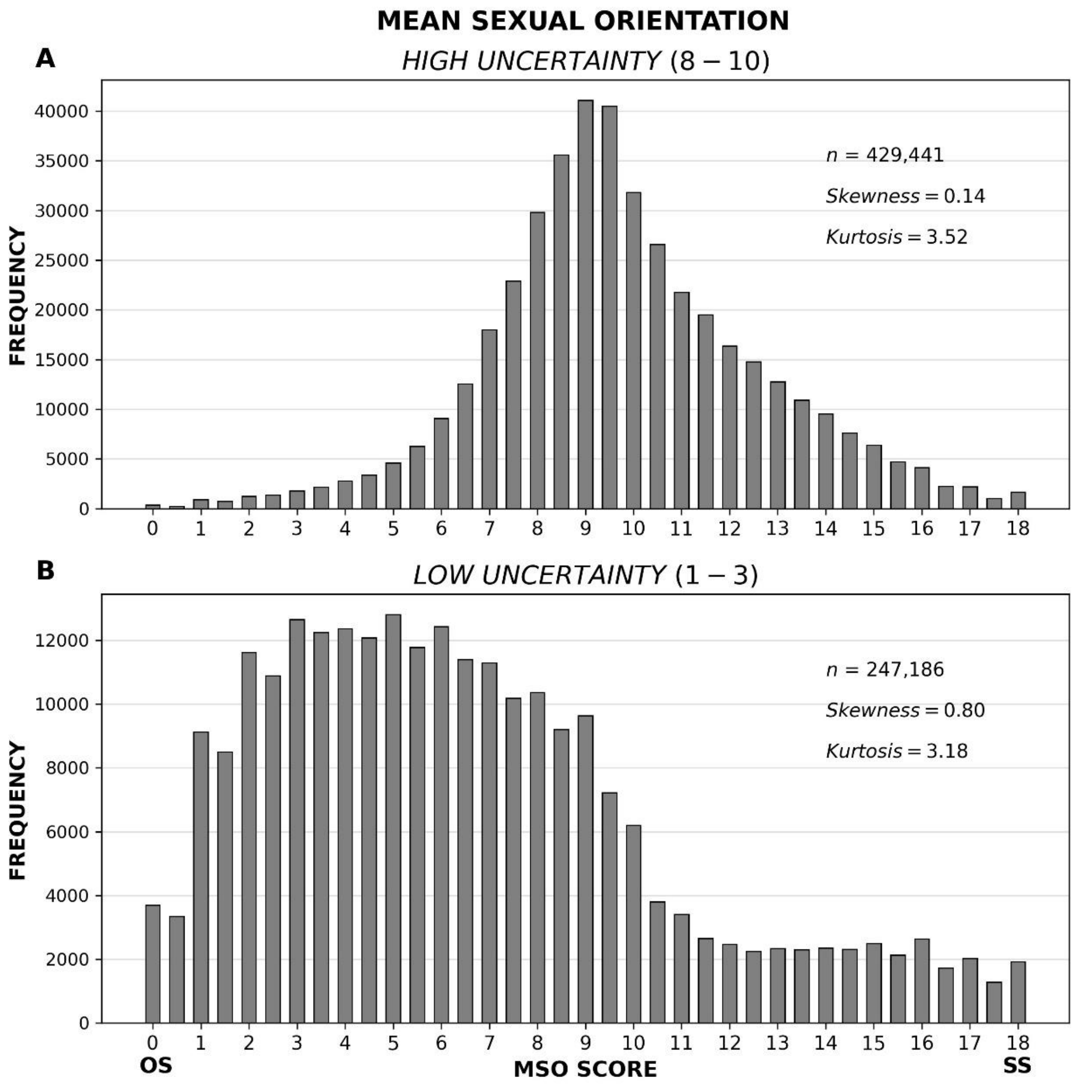

Supplementary Figure S8. MSO scores for study participants reporting low distress and low uncertainty, vs. participants reporting low distress, low uncertainty, and no change in sexual orientation.

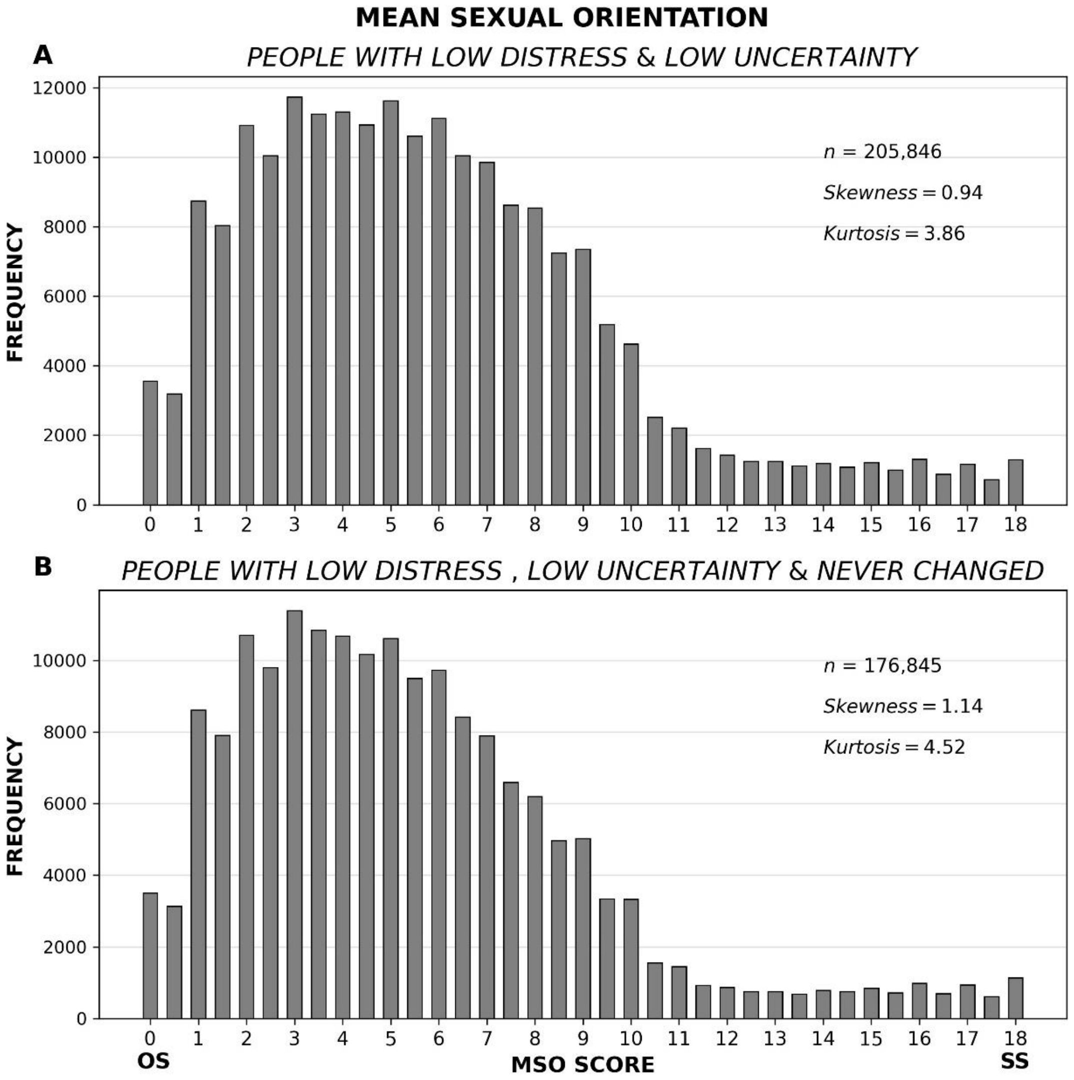

Note. Graph A shows a histogram of MSO scores for study participants reporting low distress (values 1-3) and low uncertainty (values 1-3) regarding their sexual orientation. Graph B shows a histogram of MSO scores for study participants reporting low distress (values 1-3) and low uncertainty (values 1-3) regarding their sexual orientation and also reporting that they had never changed their sexual orientation.

Supplementary Table S1. 2019 American Community Survey demographic data in the US vs. demographic data in the US in the present study.

Category	Group	2019 ACS %	Present Data %
Age	12-19	9.75	53.2
	20-34	20.4	30.3
	35-54	25.2	6.7
	55+	29.3	2.0
Race	White	60.0	68.4
	Black	12.4	5.6
	Hispanic	18.4	7.3
	Asian	5.6	10.4
	American Indian	0.7	0.8
	Other	3.0	7.4
Gender	Male	49.2	43.0
	Female	50.8	54.2
Educational Attainment	No high school diploma	11.8[a]	21.0
	High school diploma	29.8	44.4
	Some college or associate degree	36.0	4.0[b]
	Bachelor's degree and above	22.5	30.6

[a]ACS begins reporting educational attainment at age 18. The present study begins reporting educational attainment at age 12.
[b]Data in the present study do not include an option for "some college," so some of our high school graduates might belong in this group.

Supplementary Text 1. Questionnaire items.

1. Have you ever felt sexually attracted to a member of the same sex? (Possible answers: No / Yes)
2. How strongly are you sexually attracted to members of the same sex? (Possible answers: Not at all / Moderately / Very Strongly)
3. Have you ever had a dream about a sexual encounter with a member of the same sex? (Possible answers: No / Yes)
4. Have you ever had a waking fantasy about a sexual encounter with a member of the same sex? (Possible answers: No / Yes)
5. Have you ever felt sexually aroused when you've had any exposure to two people of your same gender having a sexual encounter (through gossip, a video, or some other means)? (Possible answers: No / Yes)
6. Have you ever voluntarily had sexual contact (such as kissing or petting) with a member of the same sex? (Possible answers: No / Yes)
7. Would you be willing to have sexual relations with someone of the same sex? (Possible answers: No / Maybe / Yes)
8. How frequent are your same-sex sexual fantasies or dreams? (Possible answers: Never Have Them / Rare or Occasional / Frequent)
9. How frequent are your same-sex sexual encounters? (Possible answers: Never Have Them / Rare or Occasional / Frequent)
10. Have you ever felt sexually attracted to a member of the opposite sex? (Possible answers: No / Yes)
11. How strongly are you sexually attracted to members of the opposite sex? (Possible answers: Not at all / Moderately / Very Strongly)
12. Have you ever had a dream about a sexual encounter with a member of the opposite sex? (Possible answers: No / Yes)
13. Have you ever had a waking fantasy about a sexual encounter with a member of the opposite sex? (Possible answers: No / Yes)
14. Have you ever felt sexually aroused when you've had any exposure to someone of your gender having a sexual encounter with someone of the opposite sex (through gossip, a video, or some other means)? (Possible answers: No / Yes)
15. Have you ever voluntarily had sexual contact (such as kissing or petting) with a member of the opposite sex? (Possible answers: No / Yes)
16. Would you be willing to have sexual relations with someone of the opposite sex? (Possible answers: No / Yes)
17. How frequent are your opposite-sex sexual fantasies or dreams? (Possible answers: Never Have Them / Rare or Occasional / Frequent)
18. How frequent are your opposite-sex sexual encounters? (Possible answers: Never Have Them / Rare or Occasional / Frequent)

Supplementary Text 2. Questionnaire scoring.

The questionnaire was scored as follows: Questions 1 to 9 asked about same-sex attraction, fantasies, and behavior, past and present. Questions 1, 3, 4, 5, and 6 were scored on a 0-2 scale (No = 0; Yes = 2), and questions 2, 7, 8, and 9 were also scored on a 0-2 scale (No, Not at all, Never have them = 0; Maybe, Moderately, Rare or Occasional = 1; Yes, Very Strongly, Frequently = 2). Questions 10 to 18 were the opposite-sex versions of questions 1 to 9 and were reverse scored so that questions 10, 12, 13, 14, and 15 were on a 0-2 scale (No = 2; Yes = 0), and questions 11, 16, 17, and 18 were also on a 0-2 scale (No, Not at all, Never have them = 2; Maybe, Moderately, Rare or Occasional = 1; Yes, Very Strongly, Frequently = 0). The score on each question is represented below by I*n*, where *n* is the question number:

SSRawScore = I1 + I2 + I3 + I4 + I5 + I6 + I7 + I8 + I9
OSReverseScore = I10 + I11 + I12 + I13 + I14 + I15 + I16 + I17 + I18
OSRawScore = 18 – OSReverseScore
MSO = .5 * (SSRawScore + OSReverseScore)
SOR = Lesser value (SSRawScore, OSRawScore)
SexDrive = .5 * (SSRawScore + OSRawScore)

Supplementary Text 3. Social pressure theory (SPT) of sexual orientation.

1) *Continuum.* Sexual orientation (defined as a measure of same- and opposite-sex attractions, fantasies and behaviors) exists on a continuum from exclusive opposite-sex (OS) to exclusive same-sex (SS) sexual inclinations. OS and SS inclinations can exist independently of each other.

2) *Norm.* Most people experience both types of inclinations (SS and OS) at one or more points in their lives. To put this in casual terms, everyone has a mix of "gay/lesbian" and "straight" tendencies.

3) *Social Pressure.* Heteronormativity (social pressure to express OS inclinations exclusively) suppresses the expression of SS inclinations. If a culture exists in which the pressure to express only one type of sexual inclination is completely absent, sexual inclinations in that culture will be approximately normally distributed.

4) *Bimodal distribution.* In all, or virtually all, contemporary cultures, heteronormativity suppresses SS inclinations to such an extent that (a) the distribution of sexual inclinations is skewed so that most people identify themselves as "heterosexual" when invasive surveys are used (Robertson et al. 2017), and (b) the distribution appears to break into two separate distributions, a large one consisting of people who identify themselves as heterosexual and a smaller one consisting of people whose SS inclinations are so strong they cannot be suppressed. (See below for mathematical and computational models of this process.)

5) *Fluidity*. Sexual orientation is fluid; in other words, it can change to some extent over time.

6) *Flexibility*. Sexual orientation is flexible; in other words, people can express a range of different sexual inclinations, and the magnitude of that range differs from one individual to another.

Supplementary Text 4. Theory and equations.

The mathematical formula for the social pressure theory of sexual orientation is constructed from a linear combination of two sinh-arcsinh distributions (Jones and Pewsey 2009) at certain mixture rates:

$$F_{S,p,\tau_1,\sigma_1,\mu_1,v_1,\tau_2,\sigma_2,\mu_2,v_2}(x) = p \cdot f_{\tau_1,\sigma_1,\mu_1,v_1}(x) + (1-p) \cdot f_{\tau_2,\sigma_2,\mu_2,v_2}(x)$$

Where:

$$\mu_1 = \mu_1 - exp(|S|)^2 \cdot S$$
$$\sigma_1 = \sigma_1 - |S|$$
$$v_1 = v_1 + S$$
$$\mu_2 = \mu_2 + 2 \cdot S$$
$$\sigma_2 = \sigma_2 - |2 \cdot S|$$
$$v_2 = v_2 + S$$
$$f_{\tau,\sigma,\mu,v}(x) = \frac{\tau}{\sigma} \cdot \frac{c}{\sqrt{2\pi}} \cdot \frac{1}{2\sqrt{1+z^2}} \cdot exp(-\frac{r^2}{2}))$$
$$z = \frac{x-\mu}{\sigma}$$
$$r = sinh(\tau \cdot asinh(z) - v)$$
$$c = cosh(\tau \cdot asinh(z) - v)$$

The separate distributions represent independent SS and non-SS sexual inclinations. On the sexual orientation continuum, people at the extreme low end have opposite-sex (OS) inclinations exclusively, and people at the extreme high end have same-sex (SS) inclinations exclusively. Everyone in between experiences a mixture of OS and SS inclinations. Our model shows how the overall distribution shifts and is distorted when social pressure (*S*) forbidding either SS or OS behavior is applied to the population. Currently, *S* is applied non-uniformly worldwide to push the distribution toward the OS end of the SO continuum. We define *C* as the group of people that social norms oppose – in other words, the group of people anchored at the SS of the distribution. Currently, these are people who generally self-identify as gay, lesbian, or homosexual.

All the parameters in the model were estimated from real data, specifically the dataset analyzed in the present study. The parameters are as follows:

S: Level of social pressure, S ∈ [-1,1].
p: the proportion of people in the population who self-identify as non-C.
(1 – *p*): the proportion of people in the population who self-identify as C.
μ_1: mean MSO of the population.
μ_2: mean MSO of people who self-identify as C.
τ_1: (Number of people with high certainty level (>= 8)) ÷ (Number of people with low certainty level (< 8)).
τ_2: (Number of self-identified C with high certainty level (>= 8)) ÷ (Number of self-identified C with low certainty level (< 8)).
σ_1: (Standard deviation of MSO distribution of self-identified non-C) ÷ 2
σ_2: Standard deviation of MSO distribution of self-identified C.

v_1: The Fisher-Pearson coefficient of skewness (Kokoska and Zwillinger 2000) computed from the MSO distribution of self-identified non-C.
v_2: The Fisher-Pearson coefficient of skewness computed from the MSO distribution of self-identified C.
As noted in the main body of the paper, as social pressure increases, the near-normal distribution of MSO scores becomes distorted and ultimately appears to "break" until it shows two modes, creating the impression that there are two distinct categories of sexual orientation (or, perhaps, two distinct categories of human beings). In theory, the same thing will happen if a high degree of social pressure is pushing the distribution toward the SS end of the SO continuum.

Figure S7 shows six different levels of *S* (0.0, 0.2, 0.4, 0.6, 0.8, and 1.0), generated using parameters calculated from 1.15 million cases obtained in our recent survey.

Supplementary Text 5. Main implications of SPT.

Measurement. Because OS and SS sexual inclinations can exist independently of each other, they can and should be measured separately. An individual's sexual orientation can be summarized by showing measures of OS and SS inclinations on a linear scale. The midpoint between these values – the mean sexual orientation (MSO) – is a convenient indicator of an individual's overall sexual orientation. The smaller of the OS and SS values is a reasonable estimate of an individual's sexual orientation range (SOR) (Epstein and Robertson 2014).

Labels. Sexual orientation is not a dichotomous variable, so labels such as "gay" and "straight" are simplistic. Sexual orientation is also not a categorical variable, which is why creating new labels such as "mostly gay" and "mostly straight" to try to remedy the deficiencies in the dichotomous model is at best a thankless task. Sexual orientation, like height and weight, is best characterized by numeric values on continuous ratio scales, and those values, like the values for height and weight, might change somewhat over the course of people's lives.

"Homosexuals." Although some people have strong SS sexual inclinations, the assertion – first made in the late 1800s – that such people comprise a separate and distinct class of human beings (Blank 2012) is without merit. The idea that "heterosexuals" are another separate and distinct class of human beings was introduced even later, and the so-called "bisexual" wasn't labeled until 1892 (Blank 2012; von Krafft-Ebing and Chaddock 1892). All such categorical labels should be dropped from the scientific analysis of sexual orientation.

Supplementary References

Blank, H. (2012). *Straight: the surprisingly short history of heterosexuality*. Boston: Beacon Press.

Epstein, R., and Robertson, R. E. (2014). How to measure sexual orientation range, and why it's worth measuring. *J. Bisex*. 14, 392-404. doi: 10.1080/15299716.2014.933378

Jones, M. C., and Pewsey, A. (2009). Sinh-arcsinh distributions. *Biometrika* 96, 761-780. doi: 10.1093/BIOMET%2FASP053

Kokoska, S., and Zwillinger, D. (2000). *CRC standard probability and statistics tables and formulae.* London: Chapman and Hall.

Robertson, R. E., Tran, F. W., Lewark, L. N., and Epstein, R. (2017). Estimates of non-heterosexual prevalence: the roles of anonymity and privacy in survey methodology. *Arch. Sex. Behav*. 47, 1069-1084. doi: 10.1007/s10508-017-1044-z

The United States Census Bureau. (2019a). ACS demographic and housing estimates. Available at: https://data.census.gov/cedsci/table?q=DP05andtid=ACSDP1Y2019.DP05.

The United States Census Bureau. (2019b). Educational attainment. Available at: https://data.census.gov/cedsci/table?q=Educationandtid=ACSST1Y2019.S1501.

von Krafft-Ebing, R., and Chaddock, C. G. (1892). *Psychopathia Sexualis. 7th ed*. Philadelphia: F.A. Davis Co.
